# Policy and Practice Certainty for Effective Uptake of Diffuse Pollution Practices in A Light-Touch Regulated Country

**DOI:** 10.1007/s00267-019-01242-y

**Published:** 2019-12-19

**Authors:** Jorie Knook, Robyn Dynes, Ina Pinxterhuis, Cecile A. M. de Klein, Vera Eory, Matthew Brander, Dominic Moran

**Affiliations:** 1 Business School, University of Edinburgh, 29 Buccleuch Pl, Edinburgh, EH8 9JS UK; 2 King’s Buildings, Scotland’s Rural College, West Mains Rd, Edinburgh, EH9 3JG UK; 3 Lincoln Research Center, AgResearch Ltd., 1365 Springs Rd, Lincoln, 7674 New Zealand; 4 Canterbury Agriculture & Science Centre, DairyNZ Ltd., Gerald Street, Lincoln, 7608 New Zealand; 5 AgResearch Invermay, AgResearch Ltd., Puddle Alley, Mosgiel, New Zealand; 6 The Royal (Dick) School of Veterinary Studies, University of Edinburgh, Easter Bush Campus, Midlothian, EH25 9RG UK

**Keywords:** Farmer behaviour, Water pollution, Environmental management, Advisory services, Extension

## Abstract

Although the link between agriculture and diffuse water pollution has been understood for decades, there is still a need to implement effective measures to address this issue. In countries with light-touch regulation, such as New Zealand and Australia, most efforts to promote environmental management practices have relied on voluntary initiatives such as participatory research and extension programmes; the success of which is largely dependent on farmers’ willingness and ability to adopt these practices. Increased understanding of the factors influencing farmer decision-making in this area would aid the promotion of effective advisory services. This study provides insights from 52 qualitative interviews with farmers and from observations of nine farmer meetings and field days. We qualitatively identify factors that influence farmer decision-making regarding the voluntary uptake of water quality practices and develop a typology for categorising farmers according to the factors that influence their decision-making. We find that in light-touch regulated countries certainty around policy and also around the effectiveness of practices is essential, particularly for farmers who delay action until compelled to act due to succession or regulation. The contribution of this paper is threefold: (i) it identifies factors influencing decision-making around the uptake of water quality practices in a light-touch regulated country; (ii) it develops a typology of different farmer types; and (iii) it provides recommendations on policy approaches for countries with light-touch regulation, which has potential relevance for any countries facing changes regarding their agricultural policy, such as post-Brexit policy in the UK.

## Introduction

Farm-level nitrate emissions contribute to surface and groundwater contamination (Mateo-Sagasta et al. 2017) and can be reduced by the uptake of environmental management practices. Such practices can be promoted by regulation, or in countries with light-touch regulatory approaches, they can be promoted by voluntary initiatives such as participatory extension. New Zealand provides a useful example of a country with limited government intervention in the agricultural sector, as evidenced by the level of Producer Support Estimate (PSE), which refers to gross monetary transfers from taxpayers to agricultural producers. PSE is 0.5% in New Zealand compared with 20% in countries in the European Union (OECD 2019). Due to such low policy support, initiatives stimulating the uptake of environmental practices tend to rely on the voluntary efforts of farmers (e.g. DairyNZ 2017; Kerr and Sweet 2008). Agriculture is one of the largest industries in New Zealand and nitrate leaching is currently one of the main challenges the sector is facing (Dymond et al. 2013). Communities, scientists, policy-makers and industries are pushing for change (NZ Ministry for the Environment 2017; OECD 2012), and the concept of a social licence to operate is increasingly evoked in New Zealand (Edwards and Trafford 2016). Businesses obtain this social licence when deemed legitimate, e.g. when the values of the business and its operational processes meet the expectations of local communities and other concerned stakeholders (Dare et al. 2014). This suggests that farmers need to adopt more pro-environmental practices aligned with societal expectations of good practice (Hart 2017; Legett 2017).

The New Zealand agricultural sector underwent neoliberal reform in the 1980s, when most direct and indirect government support was reduced or removed (Turner et al. 2016). Due to the light-touch approach, industry bodies are closely involved in farm practice and voluntary approaches to reduce pollution, e.g. nitrate leaching, through participatory research and extension programmes (DairyNZ 2017). Practice adoption used to be the predominant focus of the New Zealand extension models, but during the last decade there has been a change in focus by moving away from a traditional linear, technology transfer-oriented extension model into an approach where farmers become innovators, problem-solvers and co-constructors of new knowledge. These participatory research and extension activities, in which farmers, researchers and other stakeholders work together to identify good management practices (Black 2000), have been used to stimulate the voluntary uptake of water quality practices by farmers (e.g. DairyNZ 2017). Although the approach has been promoted, there are concerns about the effectiveness of this current extension design to support farmer learning about complex ideas (Sewell et al. 2017). The successful implementation of extension programmes requires an understanding of the initial phases of learning and decision-making that are important to achieve change (Turner et al. 2016). To assure effective and supportive extension more understanding is needed into the factors underlying farmer decision-making regarding the uptake of water quality practices in light-touch regulated countries.

Although Bewsell et al. (2007) show that mainly material factors, such as animal health issues or additional labour, are the main motivations for adopting water quality practices, recent studies argue that understanding behavioural change in relation to the uptake of pro-environmental practices requires a holistic approach, in which personal, material and social factors that shape the decision-making context are included (Darnton and Evans 2013; Inman et al. 2018; Mills et al. 2017; Price and Leviston 2014). Studies focusing on the uptake of water quality practices have shown that from a personal perspective, factors linked to intrinsic motivation, such as personal beliefs and norms and self-identity, are important in the adoption of these practices (Greiner et al. 2009). Personal motivations are often linked to material factors, which directly influence the productive capacity and economic viability of the farm (Burton et al. 2007; Macgregor and Warren 2006; Oreszczyn et al. 2010; Popp et al. 2007). For example a lack of financial capital is seen as a significant barrier to adoption (Yang and Sharp 2017). Another barrier is seen in labour availability, i.e. when adopting environmentally friendly practices leads to a more labour intensive system, farmers are less likely to adopt these practices (Dwyer et al. 2007). Furthermore, farmers can sometimes be wary of adopting practices, due to regular changes in legislation (Widdison et al. 2004). Social factors include farmer engagement with environmental advice, which might influence awareness and knowledge; in turn potentially influencing the ability to adopt new practices (Barnes et al. 2013; Rhodes et al. 2002). Blackstock et al. (2010) indicate how extended periods of personal interaction with experts or peers can develop trust and lead to behavioural change over time. Moreover, social capital, ‘the links, shared values and understandings in society that enable individuals and groups to trust each other and so work together’ (Keeley 2007), may also influence uptake of environmental practices by farmers who are strongly embedded in a community (Greiner and Miller 2008).

Besides focusing on a holistic decision-making model, studies have also acknowledged heterogeneity in the factors influencing farmer decision-making (Brown et al. 2016; Burton and Paragahawewa 2011). This has led to classifications of farmers based on ethnicity, class, wealth and farm size. Classifications based on these characteristics did however often not align with actual farming practice (Phillips and Gray 1995). Subsequently, studies emerged using farmer styles theory, which explains diversity in practices by using farmers’ own worldviews. It however proved difficult to identify specific farmer styles in practice (Howden and Vanclay 2000). Classifications by Barnes and Toma (2012) and Barnes et al. (2011) looked into farmer decision-making specifically around the uptake of environmental practices by applying a quantitative approach in which respondents were asked to rank predefined statements to develop a categorisation. However, these classifications only focused on perceptions, values and behaviours of farmers and did not include the relation to material factors, such as finance and farm size. A recent classification has been based on the differing extent to which individual, material and social factors influence farmer attitudes and behaviours (Mills et al. 2017), in which farmer types were inductively identified, i.e. by using primary data to identify factors, within a priori determined categories. This work was however conducted in a tightly regulated country. Qualitative work addressing farmer motivations has been done in New Zealand, but only included a subsection of the farmer population (Bewsell et al. 2007), which hinders generalisability.

Based on previous studies we identify three gaps in the current literature on farmer decision-making around diffuse pollution practices. First, most studies identifying factors influencing decision-making around the uptake of environmental practices are conducted in tightly regulated countries, such as the UK (e.g. Barnes et al. 2009; Mills et al. 2017), which leaves uncertainty around what the most important decision-making factors are in light-touch regulated countries, such as New Zealand. Second, there is only limited work focusing on how knowledge of the factors influencing decision-making and heterogeneity in these factors can be used for the design of effective extension (Brown et al. 2016; Burton and Paragahawewa 2011; Sewell et al. 2017). Third, there is a lack of inductive studies which identify generalised types or findings from the data without being guided by previous theory. The majority of recent studies have focused on identifying factors influencing farmer decision-making by using a quantitative approach in which respondents were asked to rank predefined statements (e.g. Barnes et al. 2011; Barnes and Toma 2012). In contrast, this paper applies an inductive approach to identify the factors that influence farmer decision-making in relation to the voluntary uptake of unsubsidised diffuse pollution practices in a light-touch regulated country, with the aim of using the identified factors to make recommendations on extension design. We selected the agricultural sector in New Zealand as a case study, because in recent decades, the impact of land use activities on water quality has been of increasing concern for scientists, industries, policy-makers and wider society (Roy 2019), and the New Zealand agricultural sector is characterised by light-touch regulation. Furthermore, change so far has been based on voluntary initiatives, but concerns have been raised regarding the effectiveness of current models and the extent to which these support practice-based innovation and farmer learning. Thus a better understanding of the farmers and their decision-making is crucial to inform policies that promote the uptake of good practice (Sewell et al. 2017). The contribution of this paper is threefold: (i) it applies an inductive approach to identify factors influencing decision-making around the uptake of water quality practices; (ii) it develops a farmer typology to reflect the way different types of farmers are influenced by different decision-making factors; and (iii) it provides recommendations on policy approaches and extension in countries with light-touch regulation, which has potential relevance for any countries facing changes regarding their agricultural policy, such as post-Brexit policy in the UK.

## Methods

### Study Region

Canterbury in New Zealand’s South Island was selected as our study region, because of its importance in the agricultural sector, accounting for ~20% of national agricultural land (Stats NZ 2013). In addition, it has been the location of a participatory research and extension initiative since 2013: Forages for Reduced Nitrate Leaching (FRNL) (DairyNZ 2017). This initiative involves a co-innovation approach between researchers, rural experts, and a group of farmers in Canterbury: four dairy farms; two arable farms; two sheep and beef farms; and one mixed arable and dairy farm. This group of farmers was used as an access point for observations and interviews during the study.

### Study Method

To explore farmer decision-making regarding the uptake of water quality practices we used a qualitative approach, including semi-structured interviews, meeting observations and meeting notes as our primary data. We conducted 52 in-depth face-to-face interviews with 26 dairy farmers, 10 sheep and beef farmers, 9 arable farmers, and 7 mixed farmers (Appendix 3). All interviews were recorded and fully transcribed. We also conducted observations during five meetings between farm advisors and farmers, one discussion group meeting and three field day meetings.

Appendix 1 details the interview structure, designed to provide insight into factors influencing decision-making and engagement with diffuse pollution reduction practices. The themes that were covered were: (i) background information related to the farmer and the farm; (ii) farmers’ views on nitrate leaching; (iii) nitrate management changes farmers had implemented during the last decade and farmers’ motivation behind that change; and (iv) factors that influenced farmers’ decision-making regarding the uptake of nitrate leaching measures.

### Sample Selection

To ensure the selection of a representative sample for the nitrate leaching issues in Canterbury, respondents from three groups of the Canterbury farming population were included. These respondents represented different levels of knowledge and engagement in environmental practices and extension initiatives. The first group, the ‘FRNL farmers’, consisted of farm owners and managers who were part of FRNL and thus demonstrably engaged in nitrate reduction practices. At the time of the research, monitor farms had been part of FRNL for ~4 years, during which they had gained experience in being part of a participatory research and extension programme and had been introduced to a range of practices to reduce nitrate leaching. All FRNL farmers participated in the interviews. The second group, the ‘Network farmers’, consisted of farmers who were part of the informal network of members of the first group. They were identified using snowball sampling, in which the FRNL farmers were asked to identify peers they were regularly in contact with. We conducted 18 interviews with farmers in this group. To get an overview of the decision-making factors of farmers in the network we interviewed at least one Network farmer per FRNL farm. The third group, the ‘External farmers’, consisted of farmers who had little or no involvement in environmental extension activities and had no network links. These farmers were suggested to us by key informants, also known as extension agents. The External group functioned as a ‘control’ group, assuming that these farmers had received little information via extension activities regarding nitrate leaching reduction practices. Twenty-two interviews were conducted with farmers in this group.

Five farmers rejected the invitation to participate in the interviews. Two of these farmers were Network farmers and three were External farmers. The rejection rate amongst the Network farmers was low, because FRNL farmers sent out a message to these farmers before we invited them to participate in an interview. This increased their willingness to participate. The two farmers who rejected the invitation indicated that they were too busy at the time of data collection. We do not have any additional data available on these farmers. The three External farmers who rejected the invitation were dairy farmers who were also too busy at the time of data collection. The rejection rate was low, because all External farmers had received an information email from a key informant before being invited for the interview, increasing their willingness to participate in the research. Again, apart from the contact details we did not receive any additional data on these three farmers.

The sample of farmers included in this study was intended to be representative of the nitrate leaching issues in Canterbury. Therefore, instead of representing the absolute distribution of farming types in New Zealand, the FRNL programme represented the sector according to nitrate leaching issues. This resulted in the inclusion of 56% dairy farmers, 22% sheep and beef farmers and 22% arable or mixed farmers. Consequently, the research sample consisted of 50% dairy farmers, 23% sheep and beef farmers and 27% arable or mixed farmers. The sample, with an average farmer age between 40 and 50 years old, represented the average age of New Zealand farmers, which in 2013 was 47.7 in the 2013 agricultural census (Stats NZ 2013).

### Data Analysis

The data analysis was conducted in two phases. In phase 1 the data were analysed to identify factors influencing decision-making regarding diffuse pollution reduction practices. In phase 2 we developed a typology reflecting the different factors influencing decision-making for different types of farmers.

Phase 1 analysis applied an inductive approach, drawing on Gioia et al. (2013). This sets out a systematic approach for concept development including the formation of a wide range of first order concepts before systematically placing them in second order concepts. An example of the data coding structure is attached in Appendix 2. Using content analysis software NVivo 12 (QSR International Pty Ltd 2018), we undertook an initial round of coding, using the interview data to identify first order concepts. We then undertook a second round of coding, using the data from the observed meetings, to refine and substantiate the initially identified factors, which led to the formulation of second order concepts. As part of this second round of coding, we categorised the factors under three main dimensions, *Personal*, *Material* and *Organisational*. In this study, we refer to the *Personal* dimension when we talk about farm-level influences regarding individual beliefs and attitudes and household dynamics. The *Material* dimension includes rules, regulation and infrastructure. The *Organisational* dimension is about the involvement in networks, relationships and research and extension activities.

Our inductive analysis showed early in the research process a heterogeneity in farmer decision-making, which was explained by different dominant factors in the *Personal, Material* and *Organisational* dimensions. Therefore, phase 2, developed a typology of different farmers based on the heterogeneity in decision-making factors that was observed in the dataset. To address this systematically, we attributed a ‘low’, ‘medium’ or ‘high’ weighting or a binary ‘yes’ or ‘no’ to each of the factors in the *Personal, Material* and *Organisational* dimensions to indicate the importance of the specific factor for each respondent.

## Findings

### Phase 1: Identifying Decision-making Factors

**Fig. 1:**
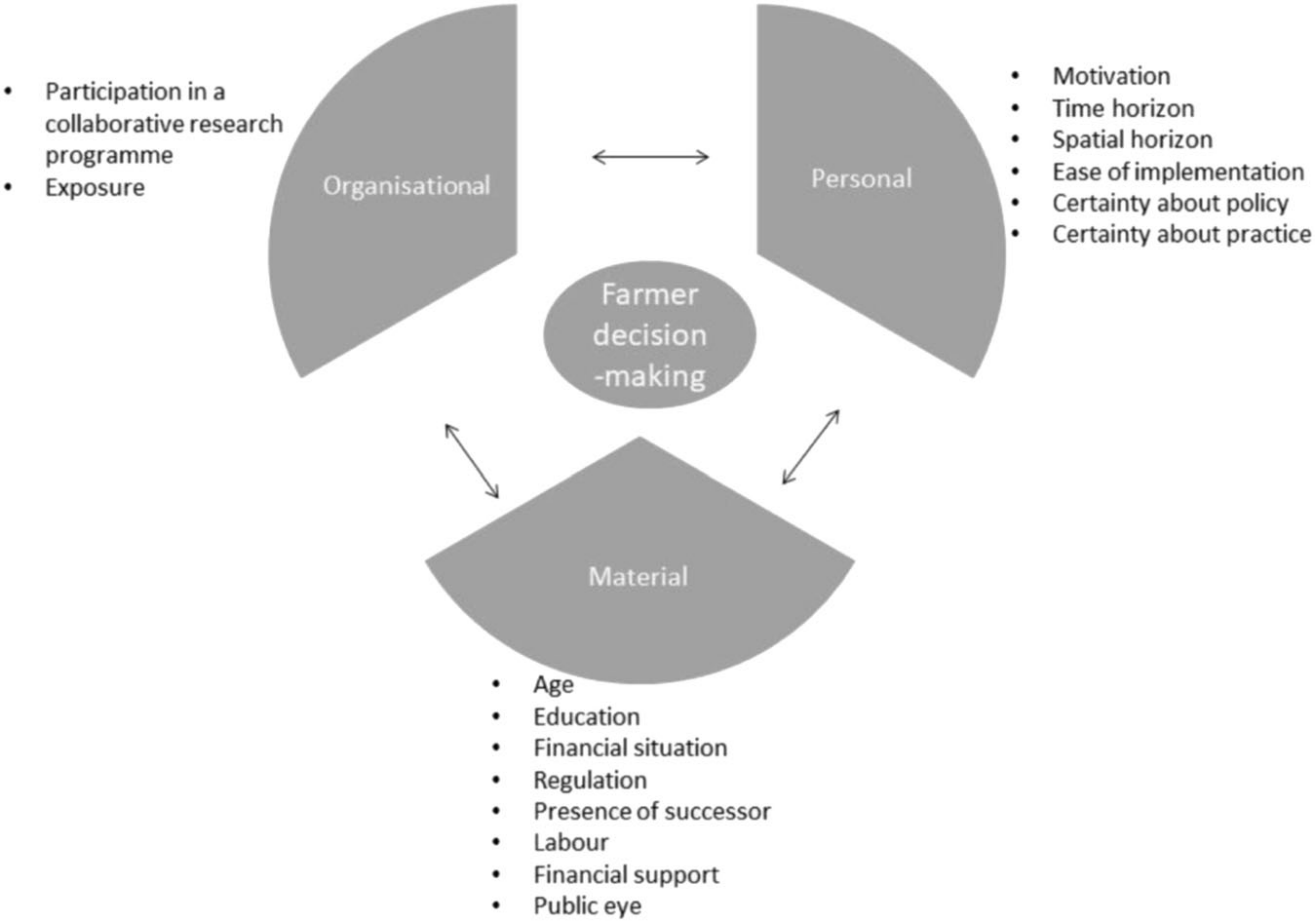
Schematic diagram of the factors influencing farmer decision-making in relation to the uptake of water quality practices

We identified a total of 16 factors influencing decision-making around the uptake of environmental practices, which we have depicted in Fig. 1. The figure indicates that these factors fall into three categories, the *Personal, Material* and *Organisational* dimensions. Furthermore, the arrows indicate the interaction between each of the dimensions.

The content analysis identified six main factors in the *Personal* dimension:

- *Motivations*: the extent to which farmers expressed intrinsic *motivations* for implementing measures. Farmers with strong intrinsic *motivations* made statements in relation to a green self-identity and personal beliefs and attitudes, such as adopting environmental measures because of ‘wanting to do the right thing’. *Motivations* were classified as ‘high’ when intrinsic *motivations* were strong, opposed to ‘low’ when intrinsic *motivations* were low.

- *Time horizon*: whether or not farmers made a connection between their current farm management and the effect it may have on future generations.
- *Spatial horizon*: the difference between farmers looking within their farm gate, versus an outward perspective, such as the catchment level or wider New Zealand society. It classifies how farmers perceived the connection between their on-farm activities and water quality issues outside the farm. The spatial horizon was ‘inward’ (as opposed to outward) when the farmer only looks within the farm boundaries.
- *Ease of implementation*: how easy farmers perceived the implementation of a new practice.
- *Certainty about policy*: whether there was certainty around the policy goals.
- *Certainty about practice*: whether there was certainty around which practices are most effective to implement.

The content analysis identified eight main factors in the *Material* dimension:

- *Age*: age of the interviewee.
- *Education*: the level of education of the interviewee.
- *Financial situation*: the extent to which the farmer experienced money to be available for investment in environmental practices.
- *Regulation*: whether there was regulatory pressure to reduce nitrate leaching.
- *Presence of a successor:* the presence of a successor to take over the farm.
- *Labour*: size of the business and amount of labour available on the farm.
- *Financial support*: the availability of financial support to implement an environmental practice. This support came from the public or private sector.
- *Public eye*: The farm was directly visible to the community, by either being located near a main road or touristic area.

From the data analysis, we identified two main factors in the *Organisational* dimension:

- *Participation in FRNL*: participation in the FRNL participatory research and extension programme.
- *Exposure*: participation in meetings other than those of a participatory research and extension programme, for example farmer discussion groups, zone committee meetings and farmer field days. Exposure was ‘high’ when farmers participated in initiatives on a regular basis. Exposure was ‘low’ when farmers did not participate in initiatives at all, or only sporadically attended meetings.

### Phase 2: Identifying Farmer Types

The observation of the trends in the importance of the factors in the *Personal, Material* and *Organisational* dimensions led us to identify five types of farmers, which reflect the difference in factors influencing decision-making. First, we noticed that there is a sub-group of farmers who showed a strong intrinsic motivation to adopt environmental practices, and who apparently have longer time horizons. However, members within this group differed regarding the financial resources and labour they had available to invest in environmental practices. Hence, this led us to create two groups of farmers with strong personal motivations, but differences in the availability of financial and labour resources. We named these farmer types the ‘*Perpetuate Cooperates*’, referring to the group with strong personal motivations and financial resources, and the ‘*Enthusiasts*’, referring to their personal enthusiasm, but acknowledging the limited availability of finance and labour. Second, we identified a type of farmer who were very business oriented, and though open to adopt environmental practices, showed a lack of intrinsic motivation. We named this type the ‘*Opportunists*’. Third, we identified a final type who were not very engaged in environmental management. However, there was still a slight difference in their outlook on adopting environmental practices, related to succession. Hence, a sub-group willing to adopt environmental practices because they felt it was necessary from a succession perspective were called the ‘*Bystanders*’. The remaining sub-group who saw no reason to get involved were called the ‘*Avoiders*’.

The type of farmer and the importance of the factors are depicted in Table 1 and the division of the interviewee groups per type is depicted in Table 2. The table does not include the *Material* factor ‘education’, because although farmers mentioned *Education* as an important factor in changing their views on water quality management, we did not specifically ask for farmers’ level of education during the interviews and thus could not analyse the influence this factor had on their decision-making.

**Table 1:** Overview of the personal, material and organisational factors constituting the types of farmers

Types	Share of farmers		Personal						Material							Organisational	
	Farmers	%	Time horizon	Focus	Intrinsic motivation	Ease of implementation	Certainty about policy	Certainty about practice	Financial situation	Presence of successor	Labour	Regulation	Age	Financial support	Public eye	FRNL	Exposure
Perpetuate Cooperates	3	6	Long	Outward	High	High	High	High	High	Yes	High	High	Low	Low	Low	Yes	High
Enthusiasts	20	38	Long	Outward	High	High	High	High	Medium	Yes/no	Medium	Medium	Average	Medium	Low	Yes	High
Opportunists	16	31	Long	Inward	Low	High	High	High	Medium	Yes/no	Medium	High	Average	High	High	No	High
Bystanders	9	17	Long	Inward	Medium	High	High	High	Low	Yes	Low	Low	Average	High	Low	No	Medium
Avoiders	4	8	Short	Inward	Low	High	High	High	Low	No	Low	Low	Average	Medium	Medium	No	Low

**Table 2:** An overview of the distribution of the interviewee groups for each of the categories

Interviewee groups	Number of farmers in group	Perpetuate Cooperates		Enthusiasts		Opportunists		Bystanders		Avoiders	
	Farmers	Farmers	%	Farmers	%	Farmers	%	Farmers	%	Farmers	%
FRNL farmers	12	2	17	6	50	4	33	–	–	–	–
Network farmers	18	1	6	6	33	4	22	4	22	3	17
External farmers	22	–	–	8	36	8	36	5	23	1	5

Although there is heterogeneity observed in the dataset, with regard to factors influencing decision-making, there were also a number of factors of importance to all farmers. First, all farmers indicated that the practices had to be easy to implement, which is for instance indicated by respondent 6:

*‘If it requires a whole lot more work, well then farmers in general are going to push back on the need to do it. If it’s easy to do than the uptake is going to be quicker and better. That’s probably just human nature really, rather than those sort of farms that are leaders of doing things like that, adopting new technologies or adopting new practices.’*

Besides ease of implementation, there was a need for certainty about policy development before implementing new practices, as illustrated by respondent 12. This interviewee indicated that he delayed implementation of changes due to uncertainty on a policy level:

*I think the way we farm here in Canterbury is going to change quite dramatically in the next probably decade or so [..] So that’s part of the reason why I'm not sort of* rushing *to make big changes just yet so that you can you know in my opinion I can see a little bit of a groundswell of change coming. I don’t want to make a big change now and then have to completely redo it again in another 5 or 10 years so—yeah, sort of just trying to buy myself a little bit of time to see where things—where the dust settles, I guess*.

This uncertainty was confirmed by respondent 37 and respondent 46. They both indicated that conflicting information is increasing the difficulty to implement changes on farm:

*‘Conflicting information is becoming more and more frustrating as we’re getting further into our farming career and we want direct, honest, accurate answers. We don’t want to be wading through a whole lot of this side and that side trying to make decisions ourselves about what’s correct. We want to be told what’s right, so that then we can try and implement* our *farm systems to suit.’*

*Noting down an answer can be quite difficult sometimes, we never quite know where we get to. [..] it’s just that, I don’t know, it’s a lot of ducking and diving and no one is held accountable. We are accountable, but people give us advice about we can do this, or that, but no one really puts a stick in the ground of what is actually supposed to happen*.

The following sub-sections provide a more detailed description of the farmer types identified.

#### Perpetuate Cooperates

Three respondents, part of the FRNL and Network farmers, fell into the category *Perpetuate Cooperates*. From the *Personal* dimension, the *Perpetuate Cooperates* had a long time horizon, an outward spatial horizon and strong intrinsic motivations. From the *Material* dimension, they had the financial resources and labour force available to enable investment in mitigation options. The *Perpetuate Cooperates* aimed to enhance employment opportunities amongst the indigenous population, and did not intend to sell their land. Respondent 1 described how these factors shape the management of the farm by talking about the bottom line:

*‘So, we’ve got financial and production, what runs the business and then we’ve got the social, cultural aspect and we’ve got the environmental and they’re the main goals, and then everything else links in between it, so yeah, they're the three main drivers that run [our business].’*

The farms of *Perpetuate Cooperates* were part of a larger business structure, which was not only active in the agricultural sector but also in other business sectors such as real estate. This broader focus gave these farmers a unique position that allowed them to invest in environmental practices. Respondent 2 emphasised this:

*‘To be fair, not all farmers are in that position [..] It’s not that you don’t want to do things, but you have to pay the bills. We’re fortunate with [our business] that we have that backing and that support to do that from day one.’*

From the *Organisational* dimension, they were highly engaged in research and extension activities, which allowed them to keep up-to-date with recent developments.

#### Enthusiasts

The largest share of respondents, 20, fell into the *Enthusiasts* category. The respondents were part of the FRNL, Network and External farmer group. From the *Personal* dimension, the *Enthusiasts* had a long time horizon, an outward spatial horizon and strong intrinsic motivations. Respondent 13 indicated that taking care of the environment is important, which is strongly related to their outward spatial horizon:

*‘We prefer to do it [implement environmental practices] because we want to. And it’s the right thing to do, not just for us, but for our wider community, as well.’*

From the *Material* dimension, this group had restricted financial resources and labour force, both mentioned as barriers to adopting environmental practices. The main difference between the *Perpetuate Cooperates* and the *Enthusiasts* can be found in this *Material* dimension. The *Perpetuate Cooperates* made more financial resources available for water quality management than the *Enthusiasts*. Due to limited financial resources, the *Enthusiast’* focus often needed to be within the farm gate, as articulated by Respondents 8 and 9:

*‘The importance is always going to be on the crop and if it’s a choice between working in some oats and sowing a commercial crop, the crop will come first. It has to. The others are not a luxury, but they are very much second best. We do it if we can.’*

*‘You know because if something is not financially viable, then any wish list you’ve got can’t be achieved. I think that’s where the environmental thing has to, I mean environmentally aware farmers are the ones that are basically making money, or can do something about it. Once they can’t make money, or they don’t have the money to spend, it does not work.’*

Both respondents talk about the trade-off between wanting to ‘do the right thing’ for the environment, versus financial imperatives. This forces the *Enthusiasts* to make a financial decision to be able to maintain a viable business, in contrast to the *Perpetuate Cooperates*, who are facing this trade-off to a lesser extent. From the *Organisational* dimension the *Enthusiasts* were highly engaged in environmental programmes.

#### Opportunists

The *Opportunists* category consisted of 16 farmers, who were part of the FRNL, Network and External farmer group. From the *Personal* dimension they were characterised by a long time horizon, an inward spatial horizon and low intrinsic motivations. From the *Material* dimension, they had sufficient financial resources and labour force available, which allowed them to adopt environmental practices. The *Opportunists* differed from the *Perpetuate Cooperates* and the *Enthusiasts* in material factors such as regulation and public perception; as is indicated by Respondent 38:

*‘Well as I say, it influences really in terms of like—as I say, if we’re going to have these lovely native plants along our roadside, we’re going to do it here where the public are, rather than doing it somewhere where no one goes.’*

Respondent 29 mentioned how he experienced public perception:

*‘It’s like farmers are like somebody in town running their business with their doors open. So it’s like leaving the board room doors open, so everybody can hear and see everything. So you get people driving past and they look at something we are doing and they make an assessment and a judgement based on their limited knowledge of, not only of agriculture, but of what is going on day-to-day on this farm.’*

This showed the farmers were aware of changes to reduce environmental impacts and were driven by the public eye in making these changes. The large size and associated high labour force of the farms allowed focus on the strategic management of the farm to identify which decisions were best from a strategic long-term perspective. From the *Organisational* dimension the farmers were highly engaged in environmental programmes.

#### Bystanders

The fourth category, the *Bystanders*, consisted of nine farmers who were part of the Network and External group of respondents. From the *Personal* dimension the farmers had a long time horizon, an inward spatial horizon and low intrinsic motivations. From a *Material* dimension, they had limited financial resources and a limited labour force that restricted them from adopting environmental practices.

From the *Organisational* dimension the farmers were not really exposed and engaged in environmental programmes, which influenced their knowledge on environmental practices. This showed a big difference between the *Opportunists* and the *Bystanders*. *Opportunists* showed high awareness of environmental regulation and the requirement for environmental protection, which they sometimes used to ‘play’ the system. However, *Bystanders* showed low awareness and had low interest in strategic decision-making. This caused them to wait longer before they made a change. This was illustrated by Respondent 46, who talked about his engagement in mitigating diffuse pollution:

*‘It would be interesting to see how that [environmental regulation] goes. I go through stages. I get quite into it and then I lose interest and I think stuff it and I sit on my hands and do nothing. A thing that I do enjoy is going out farming and growing things and watching them grow and trying things. I am quite keen to try and look after the soil and as for this side of it, it comes and goes. I will be paying attention for a while, but then it gets a bit hard, and we are not feeling like we are getting anywhere.’*

Another characteristic of this group and a main difference compared with the *Opportunists*, was the low pressure to change; they were not located in the public eye, e.g. not located near a main road or in regions subject to strict regulation.

#### Avoiders

The fifth category, the *Avoiders*, consisted of four Network and External farmers who showed a short time horizon, an inward spatial horizon and low intrinsic motivations. They were usually looking at what works on farm, and not at what happens beyond the farm gate. This was illustrated by respondent 51:

*‘I am not a politically correct type of person, I just put my head down, do my work and don’t get too involved in that kind of stuff, because it just goes over my head to be fair. I just do my thing and get on with it, until I get told I am not allowed to do it, I am just going to keep on doing it.’*

The same farm-centric view was shared by Respondent 49:

*‘I am off the radar. [..] I am aware that I perhaps should have [made an environmental plan], but over the years all I’ve ever heard is that Overseer*^1^*has had its shortcomings. Overseer has changed all the time and I haven’t felt the need to do it, because I don’t think we are leaching a lot of nutrients in the rivers. And with the jolly programme, we have to keep redoing it, so I have just paused it off. So, some of the advice is do nothing until you really have to.’*

From a *Material* dimension, this group had limited financial resources and limited labour available which restricted the adoption of pro-environmental practices. The farmers in this group were, similar to the other farmers in their awareness of public scrutiny and how it might influence their licence to operate, as illustrated by respondent 50:

*‘Any [environmental] mistake you make you can see for a long way off when you’re up in the air. So yeah, there is always that to keep on top of but yeah. [..] And if we don’t draw attention to ourselves then we’re obviously doing okay. Yeah, so that’s probably our biggest thing is to operate outside of the radar. That’s probably the goal I guess, so yeah.’*

Most of these farmers did not have a succession plan and therefore had a short-term mind-set, focused on how to maximise property value to be able to sell the property well. This was illustrated by Respondent 49:

*‘If you would have asked me [about my goals and ambitions] years ago it would have been to develop the farm into a sustainable, profitable business to be available for my children. Now to answer that question [..] I need to keep farming and set it up to be able to sell in case my children don’t want to go farming. So I guess I want to make a profit for the next 10 years from farming and then have the farm available to sell if [son or daughter] don’t wish to come home. I guess that’s where I am at.’*

## Discussion

This study shows that a combination of *Personal*, *Material* and *Organisational* factors influence farmers’ decision-making regarding the uptake of water quality practices and we have used this to derive a typology for categorising different types of farmers. The findings from the study show that in a light-touch regulated country certainty around practice and policy is essential, especially for farmers who are not likely to make changes until compelled by succession or regulation. The first part of this section focuses on the factors influencing farmer decision-making and how these factors differ to factors identified in tightly regulated countries. The second part focuses on the typology and the lessons we can draw for the design of extension programmes.

### Factors Influencing Farmer Decision-making

#### Novel decision-making factors

Farmer decision-making is known to be influenced by a wide range of factors (Inman et al. 2018; Mills et al. 2017; Waters et al. 2006). An important contribution of this study to the current literature is the identification and inclusion of the factors *Time horizon* and *Spatial horizon*, as the perception of farmers regarding these two factors has not been included in previous studies, although diffuse pollution is known to be ‘invisible’ to farmers, which affects their motivation to act upon it (Macgregor and Warren 2006). These factors are likely to be less relevant to the adoption of environmental practices that have visible outcomes, such as conservation efforts or biodiversity practices (de Snoo et al. 2013; Mills et al. 2018; Truelove et al. 2014; Van Herzele et al. 2013), but will be more relevant for practices that have seemingly distant effects (both in time and space) such as climate change (Geoghegan and Leyson 2012). The results of our study imply that communication on nitrate leaching practices needs to be tailored for farmers with short time and space horizons. A separate consideration is that although a small proportion of respondents mention *Age* as a factor, we did not find conclusive evidence of this being an influential factor in the decision-making. This finding accords with evidence in Kuehne et al. (2017).

#### The interaction of decision-making factors

Although we have provided a typology of farmers we want to highlight that farmers can change type over time, due to changes in their *Personal, Material* and *Organisational* characteristics. For example, previous research shows that focusing on financial benefits can decrease the intrinsic motivation of farmers, e.g. moving from wanting to do the ‘right thing’ to adopting practices because of financial incentives (Lokhorst et al. 2011; Van Herzele et al. 2013). Education and participation in extension programmes is also often mentioned as an important factor in influencing motivation as well. For example joint participation of researchers, farmers and experts in a participatory extension programme can promote the development of farmer self-efficacy, and this can change identities and behaviours (Sewell et al. 2017), e.g. moving farmers away from financial incentives towards the development of a green identity. This entails that interventions should not only be tailored to different farmer types, but can also aim at moving farmers from one type to another.

#### The influence of light-touch regulation

There are two material factors that limit the voluntary uptake of unsubsidised diffuse pollution mitigation practices for all farmers. First, there is the difficulty in dealing with uncertainty around policy and tools used for regulation and the effectiveness of practices. Although the regulation on nitrate leaching in New Zealand has become stricter over the last decade, in most areas there is still a high degree of uncertainty regarding nitrate leaching limits and the extent to which regulation will be enforced. Farmers indicated that lack of certainty limits investments and thus inhibits change. We suspect that farmers in a country such as the UK, which is facing a redesign of its agricultural policy, are in a similar position of dealing with uncertainty around how future support will be directed. Since we observed uncertainty to be a large decision-making factor it needs to be minimised by, for instance, setting clear mitigation targets and by assuring the effectiveness of a practice. Second, ‘ease of implementation’ is another often overlooked factor (Kuehne et al. 2017). Proposed practices often differ greatly from existing farm management and will not be adopted if there is no compensation available to overcome the costs associated with increased management or implementation complexity.

In conversations with different types of farmers we identified the importance of a social licence to operate in New Zealand. *Opportunists* often referred to this as ‘being in the public eye’ and ‘not being able to close the doors of the board room’. Dairy farmers in particular express the need to respond to increasing public scrutiny of the industry. Future research should seek to increase understanding of how it is possible to legitimise farming in a changing societal context, especially in comparison with countries where the government plays a more prominent role in the agriculture sector. Findings from the literature in organisational studies would be useful in this regard, for instance, Micelotta et al. (2017) provide an overview of possible pathways to establish and legitimise change.

### Typologies in Research and Extension

This study has presented five farmer types, which represent the differences in factors influencing decision-making related to the adoption of environmental practices. By using an inductive approach we were able to assess the range of factors mentioned by the farmers without dismissing any diverse meanings that emerged from the interviews and observations (Denzin 1971). Quantitative analysis would not have led to this depth of understanding, since a quantitative analysis would have focused on the prevalence and relationships between pre-established variables. However, we do see the value in quantitative follow-up research, which would allow us to explore whether farmers identify themselves with the suggested types. Classifying farmers has been used previously in order to target extension activities, since the influence of social, cultural, economic and physical factors on decision-making causes farmers to respond differently to encouragement to change their farming practices (Bewsell et al. 2007; Waters et al. 2006). Increasing understanding into the factors influencing decision-making and how these factors are segmented in the farmer population will help identify which extension services suit what type of farmer and which farmers to target in voluntary research and extension projects.

The next section discusses the potential lessons from our typology for the design and implementation of extension programmes. Although the typology is based on the types evident within New Zealand, it is likely that similar types, with the possible exception of *Perpetuate Cooperates*, will be present in other countries with light-touch regulation, and therefore the implications for extension design will also apply more broadly.

#### Implications for designing research and extension based on the farmer type

Extension services for the *Perpetuate Cooperates* should focus on ensuring these farmers have access to the latest knowledge and developments. However, due to their unique business structure (corporates or trusts with many shareholders or members), it is questionable whether these farmers should be targeted to set an example for the wider farming community in their region. It is also worth noting that the *Perpetuate Cooperate* type may not be common beyond New Zealand, though this should be explored in future research.

Considering the limited financial resources compared with the *Perpetuate Cooperates* it might be important for the *Enthusiasts* to create a good fit with current management practices by focusing on how to combine financial and environmental aspects in best management practice to overcome the value-action gap (Burton et al. 2008; Mills et al. 2018). On the other hand, previous research shows there is a chance that focusing on economic gain changes farmers’ decision-making from based on intrinsic motivations (wanting to do the ‘right thing’) to material factors (e.g. participating because of financial incentives) (Lokhorst et al. 2011; Van Herzele et al. 2013). Hence, emphasising the economic gain from adopting pro-environmental practices requires careful consideration, but as shown by Bewsell et al. (2007), emphasising general additional benefits, such as reduction in animal health issues or reduction in labour intensity due to a new practice, might be important to make farmers take up new practices. In addition to the framing of practices, *Enthusiasts* might benefit from education via interaction with peers and experts (Blackstock et al. 2010). For instance, via participatory programmes including a co-innovation approach, in which multiple actors from different backgrounds participate in an iterative process bringing together knowledge to support on-farm changes (Klerkx et al. 2010).

The *Opportunists* require advisory services that focus on changing motivations, so that farmers base their decision-making on intrinsic motivations instead of material factors, which supports enduring behavioural change. Changing decision-making can be achieved by participation in social groups, which can lead to the reinforcement of a ‘good farmer’ identity (Burton and Paragahawewa 2011; Mills et al. 2017; Sutherland et al. 2013). Like the *Enthusiasts*, this group would be suited to involvement in participatory programmes.

The *Bystanders* and the *Avoiders* might be difficult groups to engage with. Their lack of involvement and interest in environmental change means that current voluntary extension initiatives may be insufficient (Inman et al. 2018). Although both groups might benefit from involvement in education initiatives, it might be most efficient to provide certainty on where they need to be (e.g. through regulation or sector programmes), or to target them indirectly via peer pressure, such as ‘neighbour mimicry’ or ‘over-the-hedge farming’, in which informed farmers set an example and then have surrounding farmers take up the practice as well (Burton 2004).

Based on the characteristics of the farmers, we argue that effective extension should target different farmer types in different stages. *Enthusiasts* or *Opportunists* are the most effective to target for participatory research and extension initiatives, because they are open to new practices and their businesses show high similarity to other farms. They can therefore serve as exemplars (Brown et al. 2016). Targeting *Enthusiasts* and *Opportunists* who are surrounded by *Bystanders* and *Avoiders* could lead to mimicry or over-the-hedge farming, since these two groups of farmers are less likely to get directly involved in extension activities. Targeting the latter two groups would happen in later stages of extension, when *Enthusiasts* and *Opportunists* have successfully implemented changes. Hence, knowing different farmer types and their locations can be used to optimise engagement and can positively influence the voluntary uptake of environmental practices. With regard to identifying a farmer network as well as the types present in a certain area or network, we firstly suggest asking farmers which peers they are in touch with, as well as who their neighbours are. Consequently, types can be identified by asking farmers about their labour availability, age, education, presence of succession, whether the farm is located near roads or touristic areas and engagement in extension activities. This might be enough to create a rough categorisation and help in how to target farmers in a certain area.

#### The influence of the FRNL extension initiative

The sample for this research was based around the FRNL participatory research and extension programme. The results show that the FRNL farmers are part of the *Perpetuate Cooperates, Enthusiasts* and *Opportunists*, three groups which show high awareness of environmental practices. Considering the programme was initiated 4 years before the interviews were conducted, there is a possibility the high awareness of water quality practices and the intrinsic motivation of some of the FRNL farmers can be attributed to the extended period of personal interaction with experts and peers (Mills et al. 2008), and seeing peers involved in environmental learning (Oreszczyn et al. 2010; Sligo and Massey 2007). In future research it would be beneficial to conduct baseline interviews, to assess the progress made by farmers due to participation in such an extension programme.

Previous literature shows that verbal communication between farmers and peers is a key source of information (e.g. Oreszczyn et al. 2010; Wood et al. 2014). Based on this we expected most of the Network farmers, who are in direct contact with FRNL farmers, to show a high level of awareness and thus to fall into the *Enthusiasts* or *Opportunists* category. However, 39% of the Network farmers fell into the *Bystanders* or *Avoiders* category, which means there is a large share of farmers who are not engaging with environmental practices. Although our sample is too small to detect any significant differences, a suggestion is made by Feder et al. (2004), that this is caused by the complexity of the information, which is not easily transferred in informal (verbal) farmer-to-farmer communication. Hence, it would be helpful for designing environmental research and extension programmes to explore this in more depth, by identifying which topics are discussed within the farmer networks and whether using informal networks is an effective tool for knowledge diffusion of complex topics such as nutrient management.

## Conclusion

This paper applies an inductive research approach to identify the factors that play a role in farmer decision-making in relation to the uptake of mitigation practices for diffuse water pollution in a light-touch policy context. The typology derived from our dataset supports the view that engagement on mitigating diffuse pollution should entail a range of approaches tailored to the needs of different farmer types. Compared with studies conducted in tightly regulated countries, we identify certainty about policy and certainty about practice implementation as two highly important factors in light-touch regulated countries, because there is no subsidy available to ‘trial’ a new practice or to compensate for costs incurred by the complexity of implementing a new practice.

The identification of farmer types is of use for policy and extension design. Extensionists should identify the types of farmers present in their target area, by identifying characteristics such as their labour availability, age, education, presence of succession, whether the farm is located near roads or touristic areas and engagement in extension activities. Consequently, they can use the differences between farmers to positively influence the voluntary uptake of environmental practices via e.g. neighbour mimicry.

We suggest further research is needed into the networks of farmers participating in extension activities to gain insight into the effect of verbal communication within farmer networks. Finally, further exploration of external motivations related to ‘social licence to operate’ should be investigated, as these motivations may be increasingly important drivers for the adoption of pro-environmental practices within countries with light-touch regulation.

## Supplementary Information

Appendix 1-3

## Footnotes

1 Overseer is a software tool in New Zealand that enables farmers to look into nutrient use and farm profitability (https://www.overseer.org.nz/) and is increasingly used as a regulatory tool.
