# Supplementary material for: Policy and Practice Certainty for Effective Uptake of Diffuse Pollution Practices in A Light-Touch Regulated Country
Source: Environ Manage. 2019 Dec 19;65(2):243–56. doi: 10.1007/s00267-019-01242-y (PMC7007892; doi:10.1007/s00267-019-01242-y)
Supplement: Supplementary file 1 — Appendix 1-3 [file 267_2019_1242_MOESM1_ESM.docx]

**Appendix 1.**

An overview of the interview questions used to explore the factors influencing farmer decision-making regarding water quality issues.

*Part 1: Questions regarding background of the interviewee:*

- *Could you please describe your role on the farm?*
  - *how many hectares is the farm you are farming on?*
  - *how many employees do you have?*
  - *Optional: What is the herd size?*
- *Are you a member of a farming group? E.g. federated farmers, zone committees?*
  - *For each of the groups mentioned: how often have you met them over the past year?*
- *How would you describe your experience working on this specific farm and in the farming sector in general?*
- *How would you describe the management of the farm?*
  - *If multiple people are involved in management: who is responsible for what decision-making?*
- *How much longer do you intend to be on the property?*
  - *Do you have a successor?*
- *What are your goals/aspirations for the farm?* Brief
  - *Are these any different to what they were 5-10 years ago?*

*Part 2:*

*I would now like to specifically focus on practice changes that are known to be beneficial to the environment.*

- *Could you think of any practice changes that you have implemented since 2010 that have contributed to environmentally friendly farm practice?*
  - *Could you give any examples?*
  - *What drove you to implement these particular examples?*
- *If you contemplate on adopting an environmental practice change, what are the information sources (e.g. website, newsletter) you mostly base your decision on?*
  - *If people: what kind of background do these people have?*
  - *Do these people share your goals/aspirations for the farm?*
- *Could you describe specific challenges related to the adoption of environmental practice changes*
  - *If challenges are being identified:
    What are the causes for these challenges caused?
    How are these challenges addressed?*
- *If you decide on adopting a practice change, what are the main motivations to come to that decision? (prompt: build on an example of an earlier discussed environmental practice change)*

**Appendix 2.**

An overview of the data coding structure including quotes, first order concepts and second order concepts.

| **Example quotes** | **1^st^ order concepts** | **2^nd^ order concepts** | **Dimension** |
| --- | --- | --- | --- |
| ‘I think farmers themselves have to find a way of solving it, because they will resist and resent other people telling about it. And that's part of the reason why I have been involved in this aquifer recharge group, because it's the community itself saying 'look we have a problem and we need to find a way through it'. And also I was involved in making an input into plans that are being formed.’ | Respondent indicates the importance of community involvement | (outward) system level spatial horizon | Personal          Personal |
| ‘We’re comparing to dairy farms that winter off rather than us wintering on. So if we wintered off, you push that issue to another farm, but it’s still your issue, because it’s your cows grazing that crop. So even though we’re high here, if you drop it down, go somewhere else, you’re just taking the issue from here to somewhere else.’ | Respondent acknowledges impacts farming may have outside the farm boundaries |  |  |
| ‘There is no free rides I mean, you can't have an intensive beef system or be growing crops and putting on heaps of urea and get away with it.’ |  |  |  |
| ‘Yeah, we’re only caretakers of this land. And you lead by example. So that’s what we’re trying to do.’ | Respondent shows leadership and example |  |  |
| ‘So yes, we do need to show leadership, I think as an industry, certainly being part of [cooperation], yes we do definitely.’ |  |  |  |
| ‘Short term gain for long term pain does not work. So you want to be able to keep doing something and in a hundred years someone will be too hopefully.’ | Respondent indicates the importance of maintaining the farm in good state for future generations | (long term) time horizon |  |
| ‘Most of the farms, they think in an intergenerational way. So they are thinking about their kids grew up on this farm, and this house, hopefully one day they can take over it. And you can’t give your kids an asset that’s been devalued or polluted. We’re not thinking about that.’ |  |  |  |
| ‘I mentioned the destoning and that kind of thing, and I really think that you are only a custodian of the land and I really think that you should leave the land in a better state that you found it, if at all possible.’ | Respondent wants to leave the land in better state than it was found |  |  |
| ‘[One of the reasons we adopt environmental practices is] to leave it [the farm] in a better state than we found it.’ |  |  |  |

**Appendix 3.**

An overview of the participating farmers, their characteristics and to which type they are allocated.

| **Respondent** | **Participant group** | **Position** | **Type of farm** | **Size (ha)** | **Number of staff (incl. owner)** | **Age** | **Family run (yes/no)** | **Intergenerational (yes/no)** | **Succession (yes/no/unsure)** | **Types** |
| --- | --- | --- | --- | --- | --- | --- | --- | --- | --- | --- |
| 1 | FRNL | manager | Dairy | 353 | 4 | 20-30 | no | no | n/a | Perpetuate Cooperates |
| 2 | FRNL | general manager | Dairy | >1000 | 40 | 40-50 | no | no | n/a |  |
| 3 | Network | manager | Dairy | 304 | 4 | 20-30 | no | no | n/a |  |
| 4 | FRNL | owner-operator | Sheep & Beef | 370 | 2.5 | 50-60 | yes | no | unsure | Enthusiasts |
| 5 | FRNL | shared owner-operator | Arable | 1400 | 12 | 30-40 | yes | yes | unsure |  |
| 6 | FRNL | owner-operator | Arable | 500 | 3 | 30-40 | yes | yes | unsure |  |
| 7 | FRNL | owner-operator | Sheep & Beef | 442 | 2 | 40-50 | yes | yes | unsure |  |
| 8 | FRNL | owner-operator | Arable | 490 | 2 | 60-70 | yes | no | no |  |
| 9 | Network | owner-operator | Sheep & Beef | 320 | 2 | 50-60 | yes | no | yes |  |
| 10 | Network | owner-operator | Sheep & Beef | 1000 | 2 | 50-60 | yes | yes | unsure |  |
| 11 | FRNL | equity manager | Dairy | 540 | 9 | 40-50 | no | no | n/a |  |
| 12 | External | owner | Dairy | 230 | 5 | 30-40 | yes | no | unsure |  |
| 13 | External | owner-operator | Dairy | 928 | >6 | 50-60 | yes | yes | yes |  |
| 14 | Network | owner-operator | Dairy | 218 | 4 | 50-60 | yes | no | yes |  |
| 15 | External | Owner-operator | Dairy | 290 | 7 | 40-50 | yes | no | unsure |  |
| 16 | Network | equity-manager | Dairy | 200 | 3 | 20-30 | yes | no | n/a |  |
| 17 | Network | equity manager | Dairy | 840 | 16 | 30-40 | no | no | n/a |  |
| 18 | External | equity-manager | Dairy | 202 | 5 | 50-60 | no | no | n/a |  |
| 19 | External | owner-operator | Arable | 350 | 3 | 60-70 | yes | yes | no |  |
| 20 | Network | owner-operator | Arable | 300 | 3 | 40-50 | yes | yes | unsure |  |
| 21 | External | owner-operator | Dairy | 406 | >6 | 70-80 | yes | yes | yes |  |
| 22 | External | owner-operator | Arable | 225 | 2 | 40-50 | yes | yes | unsure |  |
| 23 | External | owner-operator | Sheep & Beef | 920 | 3 | 30-40 | yes | yes | unsure |  |
| 24 | FRNL | shared owner-operator | Dairy | 1400 | 12 | 30-40 | yes | yes | unsure | Opportunists |
| 25 | FRNL | owner-operator | Dairy | 650 | 8 | 50-60 | yes | yes | yes |  |
| 26 | FRNL | owner-operator | Dairy | 823 | 8 | 50-60 | yes | yes | yes |  |
| 27 | FRNL | manager | Dairy | 310 | 5.5 | 30-40 | n/a | n/a | n/a |  |
| 28 | Network | manager | Dairy | 314 | 4 | 30-40 | no | no | n/a |  |
| 29 | External | owner-operator | Arable | 480 | 3 | 50-60 | yes | yes | yes |  |
| 30 | Network | owner-operator | Arable | 192 | 2 | 30-40 | yes | no | unsure |  |
| 31 | Network | shared owner-operator | Sheep & Beef | 870 | 4 | 20-30 | yes | no | unsure |  |
| 32 | Network | owner-operator | Dairy | 268 | 5 | 50-60 | yes | no | n/a |  |
| 33 | External | shared owner-operator | Sheep & Beef | 930 | 4 | 40-50 | yes | yes | unsure |  |
| 34 | External | equity owner farm manager | Dairy | 338 | 5 | 50-60 | no | no | yes |  |
| 35 | External | owner-operator | Dairy & arable | 700 | 7.5 | 60-70 | yes | yes | yes |  |
| 36 | External | owner-operator | Arable | 210 | 2 | 50-60 | yes | no | unsure |  |
| 37 | External | owner-operator | Dairy | 365 | 6 | 40-50 | yes | no | unsure |  |
| 38 | External | managing director | Dairy | 1500 | >10 | 50-60 | no | no | yes |  |
| 39 | External | owner-operator | Arable | 200 | 2 | 40-50 | yes | yes | unsure |  |
| 40 | External | manager | Dairy | 398 | 5 | 30-40 | yes | yes | no | Bystanders |
| 41 | External | owner-operator | Sheep & Beef | 482 | 2 | 40-50 | yes | yes | unsure |  |
| 42 | Network | owner-operator | Dairy | 210 | 5 | 30-40 | yes | yes | unsure |  |
| 43 | Network | manager | Sheep & Beef | 6200 | 9 | 40-50 | yes | yes | n/a |  |
| 44 | External | manager | Dairy | 250 | 4 | 40-50 | yes | no | n/a |  |
| 45 | External | shared owner-operator | Sheep & Beef | 2500 | 5 | 30-40 | yes | yes | unsure |  |
| 46 | Network | owner-operator | Mixed | 360 | 2 | 30-40 | yes | yes | unsure |  |
| 47 | Network | owner-operator | Sheep & Beef | 750 | 2 | 40-50 | yes | yes | unsure |  |
| 48 | External | owner-operator | Mixed | 325 | 2 | 30-40 | yes | yes | unsure |  |
| 49 | External | owner-operator | Sheep & Beef | 630 | 3 | 50-60 | yes | yes | no | Avoiders |
| 50 | Network | owner-operator | Dairy | 130 | 3 | 30-40 | yes | no | no |  |
| 51 | Network | owner-operator | Arable | 600 | 1 | 50-60 | yes | yes | no |  |
| 52 | Network | owner-operator | Mixed | 430 | 2.5 | 30-40 | yes | yes | unsure |  |
